# Protein Folding as a Complex Reaction: A Two-Component Potential for the Driving Force of Folding and Its Variation with Folding Scenario

**DOI:** 10.1371/journal.pone.0121640

**Published:** 2015-04-07

**Authors:** Sergei F. Chekmarev

**Affiliations:** Institute of Thermophysics, 630090 Novosibirsk, Russia and Department of Physics, Novosibirsk State University, 630090 Novosibirsk, Russia; University of Leeds, UNITED KINGDOM

## Abstract

The Helmholtz decomposition of the vector field of probability fluxes in a two-dimensional space of collective variables makes it possible to introduce a potential for the driving force of protein folding [Chekmarev, J. Chem. Phys. 139 (2013) 145103]. The potential has two components: one component (Φ) is responsible for the source and sink of the folding flow, which represent, respectively, the unfolded and native state of the protein, and the other (Ψ) accounts for the flow vorticity inherently generated at the periphery of the flow field and provides the canalization of the flow between the source and sink. Both components obey Poisson’s equations with the corresponding source/sink terms. In the present paper, we consider how the shape of the potential changes depending on the scenario of protein folding. To mimic protein folding dynamics projected onto a two-dimensional space of collective variables, the two-dimensional Müller and Brown potential is employed. Three characteristic scenarios are considered: a single pathway from the unfolded to the native state without intermediates, two parallel pathways without intermediates, and a single pathway with an off-pathway intermediate. To determine the probability fluxes, the hydrodynamic description of the folding reaction is used, in which the first-passage folding is viewed as a steady flow of the representative points of the protein from the unfolded to the native state. We show that despite the possible complexity of the folding process, the Φ-component is simple and universal in shape. The Ψ-component is more complex and reveals characteristic features of the process of folding. The present approach is potentially applicable to other complex reactions, for which the transition from the reactant to the product can be described in a space of two (collective) variables.

## Introduction

Protein folding is a significant example of complex reactions, due to entropy effects and possible presence of intermediates and multiple pathways. According to the statistical viewpoint developed in the last decades of the previous century, the folding process is governed by the interplay of the protein potential energy and conformation entropy (see [1–6] for a review). The energy surface projected onto a low dimensional space of variables is funnel-like, because the effective dimension of the conformation space decreases as the protein approaches the native state, which can be explained by a hierarchical organization of the protein structure [7, 8]. The energy and entropy of the protein both decrease from the unfolded states of the protein to its native state but in different manner, so that a free energy barrier is formed that separates the native-like and unfolded states. Depending on the protein type and the conditions under which the reaction takes place, the process of folding can be complicated by the presence of on- and off-pathway intermediates and different pathways. The simplest and widely used approach to take all these factors into account, i.e., the funnel character of the energy landscape, free energy barrier, intermediates and pathways, is to construct a free energy surface (FES) depending on two collective variables [4, 9–11]. One variable is usually chosen to describe how the protein compacts as the contacts between the residues are formed (e.g., the radius of gyration), and the other how the protein conformation is close to the native state (e.g., the fraction of the native contacts). A shortcoming of the FES thus introduced is that it determines only the probabilities for the protein to be in different conformational states but does not account for the order in which these states follow each other, i.e., the local direction in which the protein proceeds. To gain a closer insight into folding dynamics, we have recently introduced a “hydrodynamic” description of the folding process [12]. In this approach, similar to the FESs, the folding process is considered in a reduced space of collective variables, but the calculated folding trajectories are used to determine local flows of transitions (the probability fluxes) in this space. Given the flows, it is possible to construct the vector field and streamlines of the folding flows, similar to how it is done in hydrodynamics [13]. If the process of folding is assumed to mimic physiological conditions, i.e., that the native state is stable and unfolding events are improbable, the folding trajectories are chosen to start at unfolded states of the protein and are terminated upon reaching the native state (“first-passage folding” [14]). In this case, the process of protein folding can be viewed as a steady flow of a folding “fluid” from the unfolded states of the protein to its native state, with the density of the fluid being proportional to the probability for the system to be at the current point of the conformation space.

The hydrodynamic approach has been successfully applied to study folding dynamics of several model proteins—an *α*-helical hairpin (a lattice model) [12], a SH3 domain [15, 16] and a *β*-hairpin protein [17] (a C_*α*_-model), and a three-stranded antiparallel *β*-sheet protein (all-atom simulations) [14, 18]. The primary conclusion drawn from these studies is that the local flow do not generally follow the FESs; even local vortices of the flows can be formed due to partial folding and unfolding of the protein that do not leave clear fingerprints on the FESs [12, 15]. It follows that the FESs do not completely govern the folding dynamics and thus can not be considered to be “true” potentials that determine the driving force of protein folding.

Recently, using a *β*-hairpin protein [19] as an example, we have shown the Helmholtz decomposition [20] of the vector field of the folding fluxes depending on two collective variables makes it possible to determine the corresponding true potential [17]. For this, the field of the probability fluxes is represented as a sum of divergence-free and curl-free fields. Accordingly, the potential has two components: one component (Φ), which corresponds to the curl-free field, is responsible for the source and sink of the folding flow that represent, respectively, the unfolded states and the native state of the protein, and the other (Ψ), which corresponds to the divergence-free field, accounts for the flow vorticity generated at the periphery of the flow field and provides the canalization of the flow between the source and sink within the reaction channel.

As such, both the hydrodynamic description of the folding dynamics and the decomposition of the vector field of probability fluxes into divergence-free and curl-free fields involve nothing that is specific of protein folding. Therefore the present approach is potentially applicable to other complex reactions that can be described by two (collective) variables, e.g., unimolecular chemical reactions [21] and conformational transitions in nanoclusters [22]. One recent example of a related approach is the calculation of a reaction path in the framework of the transition-path theory (E and Vanden-Eijnden [23]), in which the flow lines of the probability current of the reactive trajectories were introduced as the lines orthogonal to the isocommitor probability lines. The resulting pictures of the lines obtained in [23] for the model Müller and Brown potential [24] and in our work for folding of a *β*-hairpin protein [17] are very similar: the Ψ-isolines can be likened to the flow lines, and Φ-isolines to the isocommitor probability lines, although the former, in contrast to the latter, are not mutually orthogonal [17].

The *β*-hairpin protein we studied in [17] had a very simple folding scenario: a single folding pathway, no intermediates and, correspondingly, two-state kinetics. It is therefore of interest to see how the potential for the driving force changes when the folding scenario becomes more complex, i.e., involves intermediates and additional pathways. A natural approach to this problem would be to consider proteins that have the corresponding complicated scenarios and compare the results to those for the *β*-hairpin protein [17]. However, such an approach does not seem to be optimal. First, it is difficult to choose the proteins that would have the desired folding scenarios without secondary effects complicating the comparison, e.g., a protein that had parallel folding pathways and no intermediates. Secondly, the chosen proteins should allow the simulations at a reasonable computational cost, because at least the order of thousand of folding trajectories is required to have statistically significant probability fluxes [17]. This restricts the class of possible protein models to simplified, coarse-grained models, such as a C_*α*_-model used in [17]. At the same time, to describe, e.g., off-pathway intermediates, an all-atom approach is generally required, because such intermediates are typically associated with either disulfide bridges (BPTI [25]), or salt-induced states (S6 [26]), or cis prolines (CheY [27]).

Another possible approach is to use model potential energy surfaces that make it possible to generate molecular dynamics trajectories mimicking the protein folding trajectories projected onto a two-dimensional space of collective variables. These trajectories should allow both the calculation of the probability fluxes, which are necessary to determine the potential for the driving force, and the characterization of the process in terms accepted in the protein folding studies, in order to relate the potential to a specific folding scenario. For this purpose, we, similar to [23], employ the two-dimensional Müller and Brown potential [24], which is widely used to test numerical methods for calculation of reaction paths in many-body systems, e.g., [28, 29]. In addition to a simple system (one “folding” pathway without intermediates), we will consider two more complex systems: one has two folding pathways, and the other has an off-pathway intermediate. We will show that in all cases the Φ-component of the potential is simple and universal in shape, while the Ψ-component is more complex and reveals characteristic features of the folding process.

The paper is organized as follows. The next section describes methods: the generation of the PESs and their relation to protein folding, the simulation method, calculating the FESs, a short description of the hydrodynamic approach, and determining the two-component potential with the Helmholtz decomposition of the vector flow field. The subsequent section contains the obtained results and their discussion: for the basic system, a system with two folding pathways, and a system with an off-pathway intermediate. The last section summarizes the results of the work and gives concluding remarks.

## Methods

### Model potential energy surfaces and their relation to protein folding

The Müller and Brown function [24], which we use to determine a two-dimensional (2D) potential energy surface (PES), is written as

$$\begin{array}{c} U\left(x,y\right)=\sum_{i}A_{i}\exp\left[a_{i}{\left(x-x_{0i}\right)}^{2}+b_{i}\left(x-x_{0i}\right)\left(y-y_{0i}\right)+c_{i}{\left(y-y_{0i}\right)}^{2}\right] \end{array}$$ (1)

where *x*_0*i*_ and *y*_0*i*_ are the coordinates of the center of *i* potential well, *a*_*i*_, *b*_*i*_ and *c*_*i*_ characterize the steepness of the well sides, and *A*_*i*_ determines the depth of the well. The summation in this equation is taken from 2 to 4 depending on the specific form of the PES (Tables 1–3 below). Each point in the (*x*, *y*) space in Equation (1) is assumed to represent a conformation of some (imaginary) protein in a 2D space of collective variables; the coordinate *x* is to characterize the proximity of the protein conformation to the native state, and the coordinate *y*—the compactness of the protein. The deepest well on the PES is to account for the native-like states, and the shallowest well—for the unfolded states, which include both the completely unfolded (extended) and partly unfolded (semi-compact) conformations. Having this in mind, we will refer to the states of the present model system in the (*x*, *y*) space to as protein states, omitting quotation marks.

**Table 1:** Two-well landscape with a single pathway: Parameters of the generating function.

Component	*x*_0_	*y*_0_	*A*	*a*	*b*	*c*
1	0.34	0.56	-70.0	-30.0	-22.0	-12.0
2	0.75	0.2	-80.0	-40.0	-0.0	-80.0

**Table 2:** Two-well landscape with two pathways: Parameters of the generating function.

Component	*x*_0_	*y*_0_	*A*	*a*	*b*	*c*
1	0.35	0.25	-25.0	-30.0	-20.0	-10.0
2	0.65	0.2	-50.0	-50.0	0.0	-100.0
3	0.75	0.45	-25.0	-30.0	-25.0	-10.0
4	0.40	0.65	-37.5	-10.0	0.0	-10.0

**Table 3:** Three-well landscape with an off-pathway intermediate: Parameters of the generating function.

Component	*x*_0_	*y*_0_	*A*	*a*	*b*	*c*
1	0.34	0.56	-35.0	-30.0	-22.0	-9.0
2	0.75	0.15	-50.0	-40.0	-0.0	-80.0
3	0.75	0.65	-37.0	-25.0	0.0	-80.0

Two-dimensional PESs are evidently too simple to reproduce the protein folding dynamics in multidimensional conformation space. A principal shortcoming of such surfaces is that the contribution of conformational entropy is unreasonably reduced. In protein folding, when the reaction is projected onto a two-dimensional space of variables, the interplay between the potential energy and entropy leads to a barrier separating the unfolded and native-like states. On the barrier, the energy directs the protein to the native state, because its energy is minimal, but entropy pulls it back, because the number of the unfolded states is much higher that the number of native-like states (in some cases, the entropy of the native state can contribute to its stability [30], however, the overall picture of the process remains unchanged). In a 2D space, the appropriate difference in the number of the unfolded and native-like states is hardly possible to reproduce. Therefore, to mimic folding dynamics, a 2D PES should have a barrier that separates the unfolded and native-like states, although such (energy) barriers are not characteristic of protein folding. In other words, the PES should consists of, at least, two wells, one for the unfolded states and the other for the native-like states. However, if we are interested in the resulting MD trajectories rather than in the mechanism of protein folding, as in the present study, it is, in principle, insignificant what is the nature of the barrier that the system has to overcome—entropic or energetic. What is important here is that the obtained MD trajectories should allow consistent reproduction of the characteristic properties of the folding reaction in a 2D space of collective variables that are essential for the goal of the present study; they are the vector field of the local flows of conformational transitions (probability fluxes)—to calculate the potential for the driving force, and the distribution of the first-passage times and the FES landscape—to relate the results obtained with the given 2D PES to a specific folding scenario.

### Simulation method and protocols

The simulations were performed using the constant-temperature MD based on the Langevin equation

$$\begin{array}{c} \frac{d^{2}r}{dt^{2}}+\gamma \frac{dr}{dt}=-\frac{\partial U}{\partial r}+\Phi \left(t\right) \end{array}$$ (2)

where **r** is the radius-vector of the (*x*, *y*) point, *U* is the potential energy of the system given by Equation (1), **Φ** is a random force generated by the “surroundings” at temperature *T*, *γ* is the friction coefficient that introduces viscosity of the surroundings to balance the random force and dissipation, and the system’s mass is set to unity. The random forces have the Gaussian distribution with zero mean and variance ⟨Φ_*i*_(*t*)Φ_*i*′_(*t* + *τ*)⟩ = 2*γTδ*_*ii*′_ *δ*(*τ*), where the angular brackets denote an ensemble average, the index at Φ stands for the vector component, and *δ*_*ii*′_ and *δ*(*τ*) are the Kronecker and Dirac delta functions, respectively. The temperature is measured in the energy units (the Boltzmann constant *k*_B_ = 1). The equation was numerically integrated using the algorithm of Biswas and Hamann [31] with the time step Δ*t* = 0.001*τ* and *γ* = 3/*τ* (*τ* is the time scale equal to 1). In each case, 1 × 10^4^ folding trajectories were simulated at a temperature at which the folding kinetics were as desired (two-states or three states).

### Free energy surface

To construct the FES, the probability for the system to be at a current point of the (*x*, *y*) space, *p*(*x*, *y*), was calculated and then, using the Boltzmann hypothesis, was converted into the free energy

$$\begin{array}{c} G(x,y)=-T\ln p(x,y) \end{array}$$ (3)

The temperature is measured in the Boltzmann constant units.

### Probability fluxes

The probability fluxes, determining the local rates of transitions between the points of the (*x*, *y*) space, were calculated according to the hydrodynamic description of protein folding [12]. The *x*-component of the flux **j(r)** was calculated as

$$\begin{array}{c} j_{x}\left(r\right)=[\sum_{r^{\prime },r^{\prime \prime }\left(r\subset r^{*}\right)}^{x^{\prime \prime }-x^{\prime }>0}n\left(r^{\prime \prime },r^{\prime }\right)\\ -\sum_{r^{\prime },r^{\prime \prime }\left(r\subset r^{*}\right)}^{x^{\prime \prime }-x^{\prime }<0}n\left(r^{\prime \prime },r^{\prime }\right)]/\left(M{\bar{t}}_{f}\Delta y\right) \end{array}$$ (4)

where *M* is the total number of simulated trajectories, ${\bar{t}}_{\text{f}}$ is the mean first-passage time (MFPT), *n*(**r**″, **r**′) is the number of transitions from state **r**′ to **r**″, and **r** ⊂ r* is a symbolic designation of the condition that the transitions included in the sum have the straight line connecting points **r**′ to **r**″, which crosses the line *x* = const within the segment of the length of Δ*y* centered at the point **r**. The *y*-component of **j**(**r**) is determined in a similar way, except that one selects the transitions crossing the line *y* = const within the current segment Δ*x*. The calculations were performed on a grid with discretization Δ*x* = Δ*y* = 0.01.

### Potential for the driving force

To determine the potential for the driving force, the Helmholtz decomposition theorem [20] was used, similar to [17]. According to this theorem, any smooth vector field **j**(**r**) can be uniquely represented as

$$\begin{array}{c} j=j_{\mathrm{cf}}+j_{\mathrm{df}} \end{array}$$ (5)

where **j**_cf_ is the curl-free field, and **j**_df_ is the divergence-free field, i.e., ∇ × **j**_cf_ = 0 and ∇ ⋅ **j**_df_ = 0. Due to the conditions the vectors **j**_cf_ and **j**_df_ satisfy, they can be written as

$$\begin{array}{c} j_{\text{cf}}=-\frac{\partial \Phi }{\partial x}k_{x}-\frac{\partial \Phi }{\partial y}k_{y} \end{array}$$ (6)

$$\begin{array}{c} j_{\text{df}}=\frac{\partial \Psi }{\partial y}k_{x}-\frac{\partial \Psi }{\partial x}k_{y} \end{array}$$ (7)

where Φ = Φ(**r**) is the potential for the curl-free component, Ψ = Ψ(**r**) is the potential for the divergence-free component, and **k**_*x*_ and **k**_*y*_ are the unit vectors for *x* and *y*, respectively. Substituting the expressions for **j**_cf_ and **j**_df_ from Equations (6) and (7) into Equation (5) and regrouping the terms, we have

$$\begin{array}{c} j=j_{x}k_{x}+j_{y}k_{y} \end{array}$$

where

$$\begin{array}{c} j_{x}=-\frac{\partial \Phi }{\partial x}+\frac{\partial \Psi }{\partial y},\;j_{y}=-\frac{\partial \Phi }{\partial y}-\frac{\partial \Psi }{\partial x} \end{array}$$ (8)

The function Φ(**r**) and Ψ(**r**) obey the corresponding Poisson’s equations. Taking into account Equation (6) and the condition ∇ ⋅ **j**_df_ = 0, the dot product of the gradient vector and the probability flux **j** determined by Equation (5) gives

$$\begin{array}{c} △\Phi (r)+q(r)=0 \end{array}$$ (9)

where *q*(**r**) is the distribution of sources and sinks that is calculated from the simulated probability fluxes **j**(**r**) as

$$\begin{array}{c} q\left(r\right)=\partial j_{x}/\partial x+\partial j_{y}/\partial y \end{array}$$ (10)

The function Φ(**r**) should satisfy Equation (9) within a region of the **r** space in which *q*(**r**) ≠ 0 and have Φ(**r**) = 0 at its boundary (the Dirichlet problem; see, e.g., Sobolev [32]). Similarly, the cross product of the gradient vector and **j**, with taking into account Equation (7) and the condition ∇ × **j**_cf_ = 0, gives

$$\begin{array}{c} △\Psi (r)+\omega (r)=0 \end{array}$$ (11)

where

$$\begin{array}{c} \omega \left(r\right)=\partial j_{y}/\partial x-\partial j_{x}/\partial y \end{array}$$ (12)

is the simulated distribution of vorticity. As previously, the corresponding boundary condition should be satisfied, i.e., it should be Ψ(**r**) = 0 at the boundary of the region where Ψ(**r**) is determined.

To find the Φ(**r**) and Ψ(**r**), it is convenient to replace the Dirichlet problem with the initial-boundary-value problem (for the other methods of numerical solution of the former, see, e.g., Dorr [33]). In this approach, Equations (9) and (11) are replaced with

$$\begin{array}{c} \partial \Phi (r,t)/\partial t=△\Phi (r,t)+q(r) \end{array}$$ (13)

and

$$\begin{array}{c} \partial \Psi (r,t)/\partial t=△\Psi (r,t)+\omega (r) \end{array}$$ (14)

where *t* plays a role of time. In each case, starting with some initial distribution, the process is continued until a stationary distribution is achieved, which approximates the solution of the corresponding the Dirichlet problem. In the present paper, we integrated Equations (13) and (14) numerically, using a finite-difference scheme of the first-order approximation for the time derivative and the second-order approximation for the space derivatives. To avoid boundary effects, the region of integration, which was the same for Φ(**r**, *t*) and Ψ(**r**, *t*), was enlarged on each side by 0.2 in comparison with the region where the simulations were performed. The level of disretization of the **r** space was as in Equation (4). At the boundaries of this, enlarged region there was Φ(**r**, *t*) = 0 and Ψ(**r**, *t*) = 0 for Equations (13) and (14), respectively, and the initial distributions were taken to be Φ(**r**, 0) = 0 and Ψ(**r**, 0) = 0. The stationary solutions of Equations (13) and (14) were considered to be achieved if the conditions {∫[△Φ(**r**, *t*)/*q*(**r**) + 1]^2^ *dxdy*}^1/2^ ≤ 1 × 10^−10^ and {∫[△Ψ(**r**, *t*)/*ω*(**r**) + 1]^2^ *dxdy*}^1/2^ ≤ 1 × 10^−10^, respectively, were satisfied for the last one third of the total integration time. We note that Equation (8) allows determining the functions Φ(**r**) and Ψ(**r**) directly from the simulated probability fluxes **j**(**r**). For this purpose, the least-squares functional *Q* = ∫[(*j*_*x*_ + ∂Φ/∂*x* − ∂Ψ/∂*y*)^2^ + (*j*_*y*_ + ∂Φ/∂*y* + ∂Ψ/∂*x*)^2^]*dxdy*, where *j*_*x*_ and *j*_*y*_ are the simulated probability fluxes, can be minimized with respect to Φ(**r**) and Ψ(**r**) [17]. Both these approaches lead to essentially the same results.

According to Equations (6) and (7), the probability fluxes are proportional to the first-order space derivatives of the potential functions Φ(**r**) and Ψ(**r**), i.e., the fluxes are considered to be drift fluxes. In other words, although the inertia term was present in the Langevin Equation (2), the functions Φ(**r**) and Ψ(**r**) determined from the resulting simulated fluxes **j**(**r**) via Equations (5)–(7) account for an overdamped motion. The corresponding kinetic equation is the Smoluchowski equation ∂*p*/∂*t* + ∇ ⋅ **J** = 0, where *p*(**r**, *t*) is the probability density, and **J** is the probability current, which can be written as **J** = −*D*(**r**)∇*p*(**r**, *t*) + [*D*(**r**)/*T*]**F**(**r**)*p*(**r**, *t*), where −*D*(**r**)∇*p*(**r**, *t*) is the diffusion flux, [*D*(**r**)/*T*]**F**(**r**)*p*(**r**, *t*) is the drift flux, *D*(**r**) is the diffusion coefficient, and **F**(**r**) is the driving force (the Boltzmann constant is equal to unity). The zero probability current **J** = 0 assumes detailed balance, and, correspondingly, a curl-free flow. In this case, the equality **J** = 0 leads to the stationary equilibrium (Boltzmann) distribution *p*(**r**) ∼ exp[−*G*(**r**)/*T*], where *G*(**r**) is the free energy that exerts the driving force *F*(**r**) = −∇*G*(**r**). However, if the probability current is nonzero, i.e., as in the present case, when the steady flow from the unfolded states to the native state exists, the stationary solution is determined by the condition ∇ ⋅ **J** = 0, so that **J** can have a curl component (van Kampen [34]). Such flow is non-equilibrium and is characterized by “irreversible circulation” or “cyclic balance”, which can be considered as a measure of deviation from detailed balance [35–37]. In the present case, the circulating flow is represented by the flux vector **j**_df_ and, according to Equation (7), is generated by the Ψ-component of the potential.

We note that the functions Φ(**r**) and Ψ(**r**) in Equations (6) and (7) differ from the conventional potentials in that the driving forces determined by these functions are density weighted. Conventionally, as, e.g., in the Smoluchowski equation above, the probability flux is determined as **j**(**r**) ∼ **F**(**r**)*p*(**r**), so that the driving force **F**(**r**), which is equated with the negative gradient of the potential, is proportional to the velocity of motion **v**(**r**) = **j**(**r**)/*p*(**r**). In contrast, Equations (6) and (7) assume that the gradients of the potentials are immediately related to the probability fluxes. The reason why the conventional definition is unsuitable in the present case is that the probability density *p*(**r**) varies across the flow field very strongly, i.e., the folding fluid is highly “compressible”. Because of this, as it follows from the equality ∇ ⋅ **v**(**r**) = ∇ ⋅ **j**(**r**)/*p*(**r**) + **j**(**r**) ⋅ ∇[1/*p*(**r**)], the velocity field is not divergence-free in a region which is free of flow sources and sinks, in contrast to the field of the probability fluxes, for which ∇ ⋅ **j**(**r**) = 0. The last term in the right-hand side of the above equality presents the sources/sinks that arise due to the compressibility of the folding fluid. The appearance of such artificial sources and sinks destroys the natural separation of the folding flow into the divergence- and curl-free components and complicates the interpretation of the Φ(**r**) and Ψ(**r**) functions (see below). Therefore, we determine the potential through the fluxes, according to Equations (6) and (7), but consider the driving force to be density weighted, i.e., $\text{F}(\text{r})=p(\text{r})\tilde{\text{F}}(\text{r})$, where $\tilde{\text{F}}(\text{r})$ is the actual driving force.

## Results and Discussion

### Two-well landscape with a single pathway

**Fig 1:**
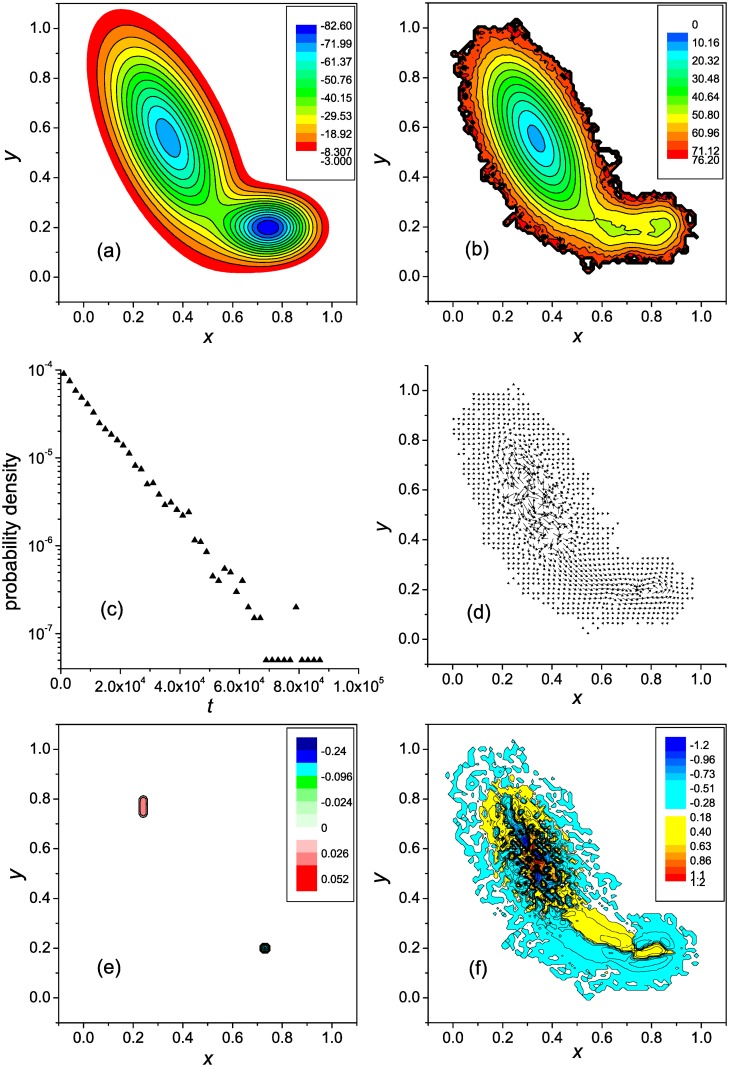
Two-well landscape with a single pathway: General characterization. (**a**) the potential energy surface, (**b**) the free energy surface, (**c**) the first-passage time distribution, (**d**) the vector flow field, (**e**) the distribution of the divergence, and (**f**) the vorticity distribution.

This case corresponds to the simplest, two-state kinetics of protein folding, which are characteristic of small proteins [38]. Table 1 gives the values of the parameters determining the potential energy function, Equation (1), and Fig. 1**a** shows the corresponding PES. The simulations were performed at *T* = 3.0. The native state was associated with the point *x*_n_ = 0.75, *y*_n_ = 0.2. The MD trajectories were initiated in the vicinity of the point *x*_u_ = 0.25, *y*_u_ = 0.78 with a uniform random scattering of the points within ∣Δ_u_ *x*∣ = ∣Δ_u_ *y*∣ = 0.1; these points were intended to mimic the completely unfolded (extended) protein states. The native state was considered to be reached if the deviation from the native state was not larger than ∣Δ_n_ *x*∣ = ∣Δ_n_ *y*∣ = 0.01. The same conditions to choose the initial points (the scattering of the points) and to terminate the trajectories in the native state were used for the other systems.

Fig. 1**b** shows the FES. It reveals two basins of attraction that are connected by a free energy valley, which plays a role of the reaction channel. One basin, which is located at lower values of *x*, can be associated with unfolded conformations. For the most part, these conformations should be considered as partly unfolded (semi-compact), because extended conformations rapidly transform into semi-compact ones [11]. The other basin corresponds to native-like conformations. Since the trajectories were terminated upon reaching the native state, this basin is not so well formed as it would be under the equilibrium folding conditions, when the protein resides in the native basin until it unfolds (cf., e.g., the FESs for the first-passage and equilibrium folding of the three-stranded *β*-sheet protein [14]). At the same time, the free energy barrier between the unfolded and native-like states is rather well formed (*x* ≈ 0.57).

The distribution of the first passage times is depicted in Fig. 1**c**. The distribution is close to single-exponential, evidencing that the kinetics are two-state. The MFPT ${\bar{t}}_{\text{f}}$ is equal to ≈ 1 × 10^4^.

Fig. 1**d** shows the vector field of the probability fluxes. For the illustration purpose, the lengths of the vectors are rescaled as **j**_scl_ = *q*(**j**/*j*)*j* ^1/4^, where **j** is the original vector determined by Equation (4), *j* is its length, and *q* is the factor equal to 0.125. As is seen, the complex motion of the system that exists in the basin associated with unfolded states changes to a regular motion in the transition region between this and native basins, i.e., in the region which contains the free energy barrier. In this respect, the present vector flow field is similar to what was observed for an *α*-helical hairpin, which also had a single folding pathway and two-state kinetics [12]. A vortex observed in the native basin is mainly due to the strong condition we used to terminate the trajectories (∣Δ_n_ *x*∣ = ∣Δ_n_ *y*∣ = 0.01). Because of it, the system had to explore a large portion of the native-like states until the native state was found with the required accuracy. For actual protein folding, when the multidimensional conformation space is projected on a two-dimensional space of collective variables, this effect is not present [12, 17]. If the values of Δ_n_ *x* and Δ_n_ *y* were taken to be comparable to the size of the native basin (∣Δ_n_ *x*∣ = ∣Δ_n_ *y*∣ ∼ 0.1), the vortex was not formed. Instead, a region around the native state was created, into which the trajectories did not penetrate, i.e., the trajectories ended at the boundary of this region. We also note that since every trajectory initiated in an unfolded state reached the native state, and was terminated in it, the total flow from the unfolded states to the native state *G*(*x*) = ∫ *j*_*x*_(*x*, *y*)*dy* is constant; it is equal to the reciprocal MFPT $1/{\bar{t}}_{\text{f}}$ [see Equation (4)].

Fig. 1**e** and 1**f** show, respectively, the distributions of the flow divergence *q*(**r**) and its vorticity *ω*(**r**), which are determined by Equations (10) and (12). Similar to the probability fluxes (Fig. 1**d**), for the illustration purpose, the values of *ω*(**r**) are rescaled as *ω*_scl_ = (*ω*/∣*ω*∣)∣*ω*∣^1/4^. Fig. 1**e** shows that except for the local regions where the trajectories were initiated and terminated, i.e., the source and sink regions, the flow is divergence-free. The vorticity, in contrast, is nonzero across the entire flow field (Fig. 1**f**). First of all, it is seen that the motion of the system in the basin for the unfolded states is not completely disordered—a set of the vortices of opposite sign exists there (the negative vorticity corresponds to the clock-wise motion, and the positive vorticity to the counterclockwise motion). Secondly, the vorticity has different signs on different sides of the reaction channel, because the intensity of the flow decreases toward both sides of the channel (Fig. 1**d**). As has been mentioned in [17], this decrease of the flow intensity toward the channel sides is a natural phenomenon, because the lower the probability to visit some region of the conformation space (in the present case, a side of the channel), the smaller the flows in this region; similar nonuniform distributions of the flows were previously observed for an *α*-helical hairpin [12], SH3 domain [15], and *β*-hairpin [17]. Therefore, the vorticity of this type is an intrinsic property of the folding dynamics.

**Fig 2:**
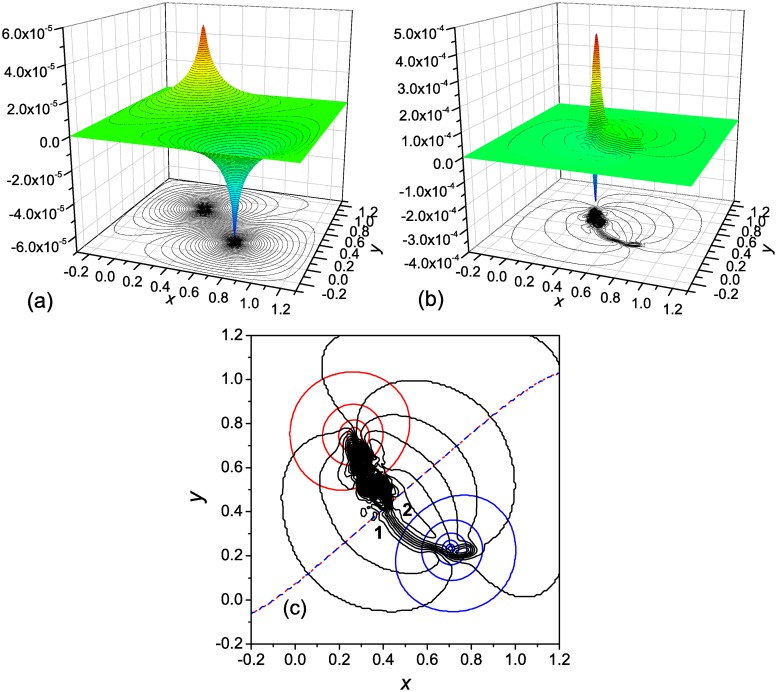
Two-well landscape with a single pathway: The potential for the driving force. (**a**) the Φ-component of the potential, (**b**) the Ψ-component, and (**c**) the isolines Φ(**r**) = const (blue and red lines are, respectively, for the negative and positive values, and the blue-red dashed line is for the zero value), and Ψ(**r**) = const (black lines). In panel **c** the Φ(**r**) = const and Ψ(**r**) = const isolines are shown with interval of $0.1/{\bar{t}}_{\text{f}}\approx 1\times 10^{-5}$ (see the text). Characteristic values of Ψ(**r**) in the reaction channel: in region 1 Ψ(**r**) ≈ −2 × 10^−5^, and in region 2 Ψ(**r**) ≈ 5 × 10^−5^.

The calculated functions Φ(**r**) and Ψ(**r**) are shown in Fig. 2**a** and 2**b**, respectively. In agreement with the Helmholtz decomposition [20], Φ(**r**) accounts for the source and sink of the folding flow, and Ψ(**r**) for the vorticity effects. The surface for the Φ-component is simple; it is characterized by two peaks of different sign—one (positive) peak corresponds to the source of the folding flow, and the other (negative) peak represents the sink of the flow. Poisson’s Equation (9), in which *q*(**r**) > 0 in the source region, *q*(**r**) < 0 in the sink region, and *q*(**r**) = 0 in the rest of the **r** space, can be viewed as an equation that describes the stationary diffusion of some substance of density Φ(**r**) between the source and sink. It is evident that the substance ejected from the source will spread over the surface in all directions. Therefore, to canalize the flow between the source to sink, an addition force is required. This force is produced by the Ψ-component of the potential. Two properties of the Ψ(**r**) surface are noteworthy. The first one is that in the region between the source and sink, where the Φ(**r**) surface is relatively flat, two parallel ridges of different sign are formed that connect the source and sink. More clearly, it is seen from Fig. 2**c**, where characteristic values of the Ψ-component in the regions corresponding to the ridges are indicated: the Ψ-component decreases from the periphery of the flow field, where it is zero (Fig. 2**b**), to approximately −2 × 10^−5^ in region 1 (the first ridge) and then increases to 5 × 10^−5^ in region 2 (the second ridge), with a subsequent decrease to zero at the opposite side of the flow field. The second property of the Ψ(**r**) surface is the presence of (two) peaks of different sign in the region for the unfolded states, which are due to the local vortices formed in this region (Fig. 1**f**). Such vortices were observed for the *α*-helical hairpin [12], SH3 domain [15] and three-stranded *β*-sheet protein [14], as a result of partial folding and unfolding of the protein in the attempts to overcome the barrier to the native state. In should be noted that in contrast to the presence of parallel ridges of different sign in Ψ(**r**), which is typical of the folding process [17], the appearance of the peaks in Ψ(**r**) depends on the value of the free energy barrier between the unfolded and the native states. If the barrier is large enough, the system dwells in the basin for the unfolded states performing a circulating motion, as in the present case; then the peaks are formed. However, if the barrier is low, so that the system easily overcomes it, the peaks are absent [17].

Fig. 2**c** depicts the equipotential lines Φ(**r**) = const and Ψ(**r**) = const. In each family, the lines are shown with the interval of $0.1/{\bar{t}}_{\text{f}}$. In the intermediate region between the source and sink, where the Φ(**r**) surface is relatively flat, the Ψ(**r**) isolines are directed along the probability fluxes [17], i.e., each of them presents a streamline of the flow. Every two adjacent streamlines form a stream tube, so that the (ten) stream tubes between the Ψ(**r**) isolines in this region contain the total flow from the source to the sink [see Equation (4)]. As has been mentioned in the Introduction, in contrast to [23], in which the probability current lines (streamlines) were introduced as the lines orthogonal to the isocommitor probability lines, the sets of the Φ(**r**) = const and Ψ(**r**) = const lines are generally not mutually orthogonal because the folding flow is not curl-free; the orthogonality of these lines would require the flow to be both divergence- and curl-free [13] (see the discussion of this issue in [17]).

As has been previously indicated, the velocity field **v**(**r**) = **j**(**r**)/*p*(**r**) for a compressible folding fluid, i.e., for the probability density *p*(**r**) ≠ const, is not divergence-free. The calculations show that if the velocities are used instead of the fluxes in Equations (6) and (7), the Φ and Ψ components of the potential presented in Fig. 2 change very considerably. The main reason is that the probability density *p*(**r**), which has a large value in the basin for the unfolded states, drastically decreases toward the native state. As a result, the velocity of the flow **v**(**r**) increases, and the effective source of the flow, determined by the velocity, shifts toward the flow sink. Correspondingly, the positive peak in Φ(**r**) shifts toward the negative peak, and the ridges between the source and sink regions in Ψ(**r**) become considerably shorter. In other words, both Φ(**r**) and Ψ(**r**) surfaces lose their direct connection with the physical picture of the process.

### Two-well landscape with two pathways

**Fig 3:**
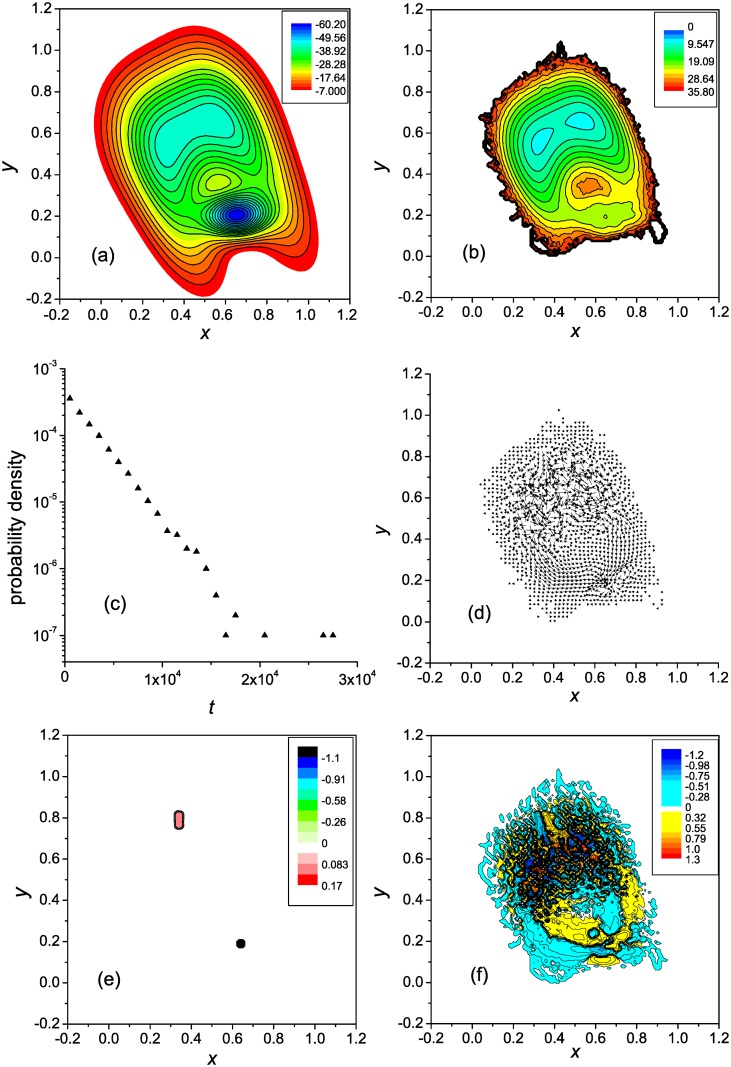
Two-well landscape with two pathway: General characterization. Notations are as in Fig. 1.

**Fig 4:**
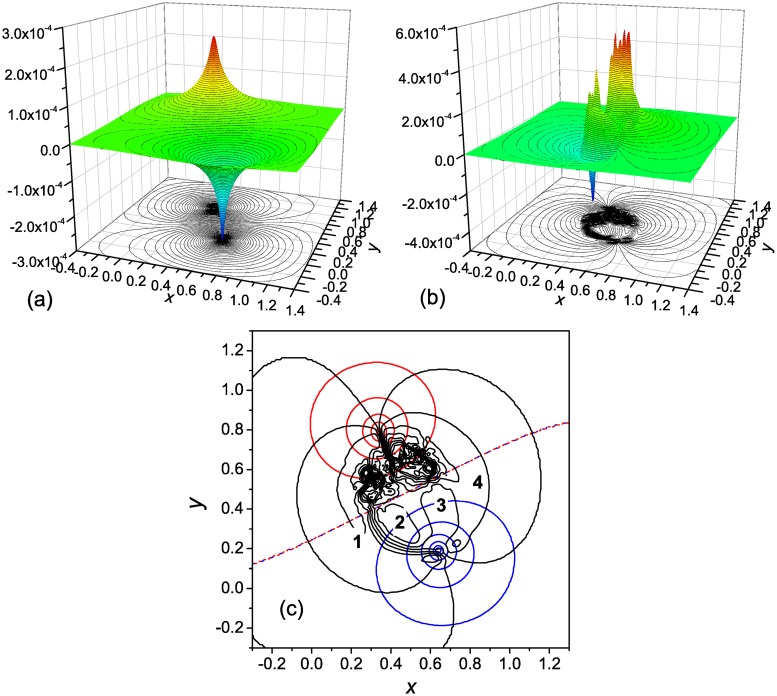
Two-well landscape with two pathway: The potential for the driving force. General notations are as in Fig. 2. In panel **c** the Φ(**r**) = const and Ψ(**r**) = const isolines are shown with interval of $0.1/{\bar{t}}_{\text{f}}\approx 4.5\times 10^{-5}$. Characteristic values of Ψ(**r**) in the reaction channel: Ψ(**r**) ≈ −1 × 10^−4^ (region 1), Ψ(**r**) ≈ 1.3 × 10^−4^ (region 2), Ψ(**r**) ≈ 3.8 × 10^−5^ (region 3), and Ψ(**r**) ≈ 8.2 × 10^−5^ (region 4).

One complication of the folding scenario described in the previous section is the presence of multiple folding pathways [39, 40]. We will consider the simplest case when there are two independent pathways, as, e.g., in the B domain of protein A due to the symmetrical backbone structure of the protein (a three-helix bundle) [41, 42], or in the fyn SH3 domain, where the fast folding trajectories are organized in two essentially independent routes due to the reverse order of formation of the contacts between the *β*1 and *β*5 strands and the RT-loop and the *β*4 strand [15]. The values of the parameters determining the potential energy function in the present case are given in Table 2, and the corresponding PES is shown in Fig. 3**a**. The temperature at which the simulations were performed was *T* = 1.5. The native state was associated with the point *x*_n_ = 0.65, *y*_n_ = 0.2, and the MD trajectories were initiated in the vicinity of the point *x*_u_ = 0.35, *y*_u_ = 0.8. The MFPT is ${\bar{t}}_{\text{f}}\approx 2.2\times 10^{3}$. Figs. 3**b**-3**f** and 4**a**-4**c** show the simulation results, akin to the corresponding panels of Figs. 1 and 2.

According to Fig. 3**a** and 3**b**, the right-hand pathway from the unfolded to the native state is more effective (see also Fig. 3**d**). Since the pathways are independent, the kinetics remain two-state [43] (Fig. 3**c**), as it were in the case of a single pathway (Fig. 1**c**). Also, similar to the single pathway (Fig. 1**f**), each of the pathways is characterized by the change of the flow vorticity from negative to positive across the corresponding reaction channel. Accordingly, two pairs of parallel ridges are formed that connect the source and sink regions (Fig. 4**b**). Some details are given in Fig. 4**c**. The function Ψ(**r**) first decreases from zero at the periphery of the flow field (Fig. 4**b**) to approximately −1.4 × 10^−4^ (region 1) and then increases to 1.3 × 10^−4^ (region 2), which represents the first pair of ridges. This is followed by formation of the second pair of ridges, in which Ψ(**r**) decreases to 3.8 × 10^−5^ (region 3) and then increases to 8.2 × 10^−5^ (region 4). The peaks in the Ψ-component are of the same origin as for the single pathway PES (Fig. 2**b**). Despite the dynamics of the system in the present case, and the Ψ(**r**) surface as well, are more complex than in the case of the single pathway, the Φ-component (Fig. 4**a**) is of the same shape as in the latter case.

### Three-well landscape with an off-pathway intermediate

**Fig 5:**
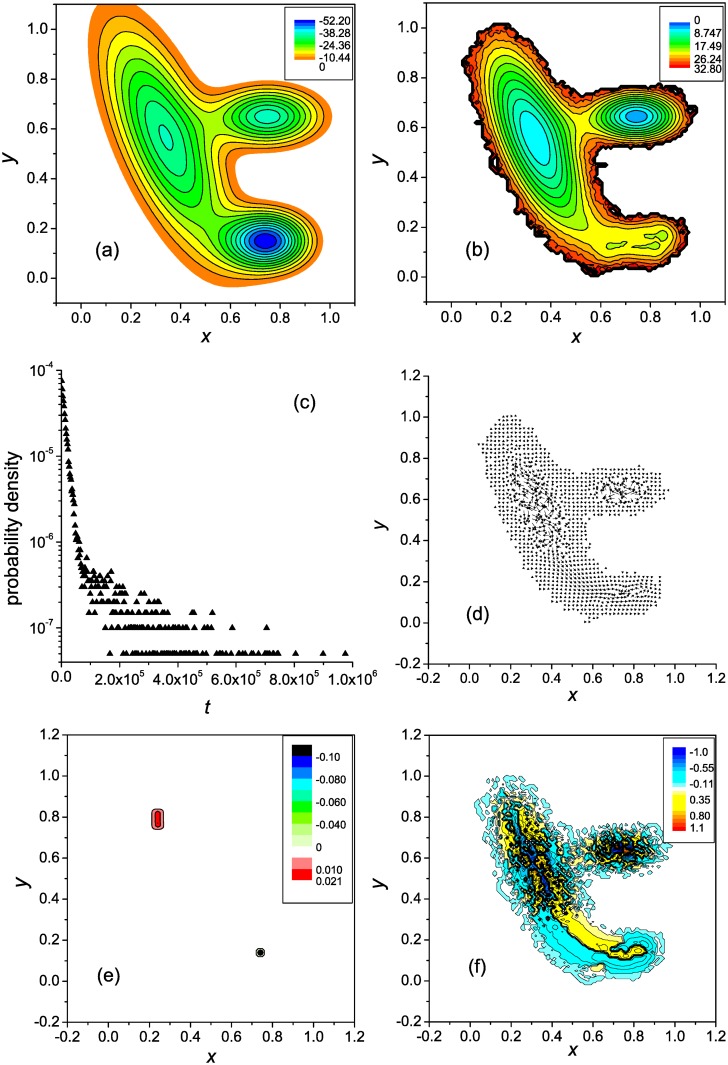
Three-well landscape with an off-pathway state: General characterization. Notations are as in Fig. 1.

**Fig 6:**
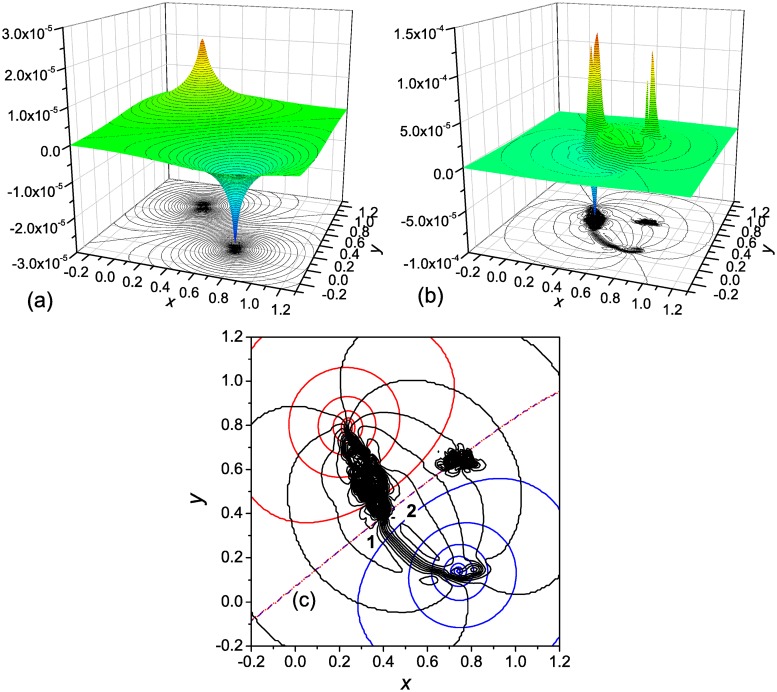
Three-well landscape with an off-pathway state: The potential for the driving force. General notations are as in Fig. 2. In panel **c** the Φ(**r**) = const and Ψ(**r**) = const isolines are shown with interval of $0.1/{\bar{t}}_{\text{f}}\approx 4\times 10^{-6}$. Characteristic values of Ψ(**r**) in the reaction channel: in region 1 Ψ(**r**) ≈ −1 × 10^−5^, and in region 2 Ψ(**r**) ≈ 2 × 10^−5^.

Another typical complication of the basic folding scenario described previously is the presence of off-pathway intermediates, which lead to a deviation from two-state kinetics [11]. When comparable with the native state in the life-time, such intermediates can play a role of “latent” states [44]. Table 3 gives the parameters of the potential energy function we used to construct the model PES in this case, and Fig. 5**a** depicts the PES. The simulations were performed at *T* = 1.25. The native state was associated with the point *x*_n_ = 0.75, *y*_n_ = 0.15, and the MD trajectories were initiated in the vicinity of the point *x*_u_ = 0.25, *y*_u_ = 0.8. The off-pathway intermediate is presented by a basin centered at *x* = 0.75, *y* = 0.65. The MFPT is ${\bar{t}}_{\text{f}}\approx 2.5\times 10^{4}$. The simulation results are shown in Figs. 5**b**-5**f** and 6**a**-6**c**.

In accord to the FES (Fig. 5**b**), Fig. 5**c** indicates that the kinetics are double-exponential; the MFPTs for the fast and slow trajectories are equal to approximately 1.5 × 10^4^ and 1.5 × 10^5^, respectively. In the fast trajectories, the system goes from the unfolded states to the native state directly, while in the slow trajectories, it visits the intermediate (possibly, several times) before proceeding to the native state. The directed motion toward the native state is accompanied by a gradual change of the flow vorticity across the reaction channel (Fig. 5**f**), similar to what was observed for the systems considered in the previous sections. Accordingly, a pair of parallel ridges is formed that connect the source (unfolded states) and the sink (native state) of the flow (cf. Figs. 6**b** and 5**d**). At the same time, the reaction channel between the unfolded states and the intermediate does not possess these properties, because the flow toward the intermediate is compensated by the backward flow. Correspondingly, the intermediate presents neither a source nor sink of the flow, which is reflected in the distribution of divergence (Fig. 5**e**) and the Φ-component (Fig. 6**a**), and the Ψ(**r**) surface does not have ridges connecting the unfolded states and the intermediate (Fig. 6**b**). Also note that a set of vortices is formed in the basin associated with the intermediate (Fig. 5**f**), similar to the basins for the unfolded states in this (Fig. 5**f**) and previous systems (Figs. 1**f** and 3**f**). These vortices lead to formation of a set of peaks of positive and negative sign that the Ψ-component has in the intermediate basin.

## Conclusions

Using model potential energy surfaces generated with the 2D Müller and Brown potential [24], three characteristic scenarios of protein folding have been considered: a single pathway from the unfolded to the native state without intermediates (two-state kinetics, a basic scenario), two parallel pathways without intermediates (two-state kinetics), and a single pathway with an off-pathway intermediate (three-state kinetics). It has been shown that despite a reduced contribution of conformation entropy, the 2D surfaces enable consistent reproduction of the characteristic properties of the folding reaction in a 2D space of collective variables that are essential for the goal of the present study, i.e., the vector field of the probability fluxes, which is used to calculate the potential for the driving force, and the distribution of the first-passage times and the FES landscape, which are necessary to relate the results obtained with the 2D PES to a specific folding scenario. To calculate the probability fluxes, the hydrodynamic description of the folding reaction [12] was employed. In it, the process of the first-passage folding is viewed as a steady flow of the representative points of the system from the unfolded to the native state. One essential property of the hydrodynamic approach is that the Helmholtz decomposition [20] of the vector field of the probability fluxes makes it possible to introduce the potential for the driving forces of folding [17]. This potential has two components, Φ(**r**) and Ψ(**r**); the Φ-component accounts for the sources and sinks of the folding flows, and the Ψ-component for the flow vorticity effects. Both components obey Poisson’s equations; in the equation for Φ(**r**), the source/sink term represents the distribution of the flow sources and sinks, and in the equation for Ψ(**r**), the distribution of the flow vorticity.

The consideration of the above mentioned scenarios has led us to the following conclusions: Despite all possible complexity of the folding process, the Φ-component of the potential is simple and universal in shape. It has two peaks of different sign; one (positive) peak corresponds to the source of the folding flow, representing the unfolded states in which the folding trajectories are initiated, and the other (negative) peak corresponds to the sink of the flow, representing the native state, where the trajectories are terminated. The Ψ-component is more complex and depends on the specificity of the folding process. The most notable property of the Ψ(**r**) surface is the presence of parallel ridges of different sign that connect the source and sink of the flow; if there are several independent pathways, the corresponding pair of such ridges is formed for each of them. These ridges are the result of the flow vorticity, which gradually changes across the free energy valley connecting the unfolded and native states (the reaction channel) and has different signs at the sides of the valley, because the folding flow weakens toward both sides. The components Φ and Ψ act in concord, i.e., in the region between the source and sink they are such that the fluxes generated by these components contribute to the directed flow from the source to the sink, while in the regions behind the source and sink they compensate each other. In the region between the source and sink, where the Φ(**r**) surface is relatively flat, the Ψ-component plays a primary role, providing the canalization of the flow within the reaction channel. Another, although not so characteristic [17], property of the Ψ(**r**) surface is the possible presence of peaks of different sign in the regions where the system dwells performing a circulating motion, as it happens in the basins for the unfolded states and intermediates. The formation of these peaks depends on the heights of the barriers to leave these basins, i.e., if the system leaves the basin easily, the peaks are not formed.

For a system with many degrees of freedom, the present potential reflects the complexity of the reaction only in the part that is captured by two collective variables. For protein folding, the practice of application of the principal component analysis and other methods of dimensionality reduction (for a review, see, e.g., [18]) suggests that the general picture of the reaction, i.e., the dominant folding pathways and long-living intermediates, will likely be captured in a two-variable description. At the same time, some specific features of the reaction, which require a more detailed characterization of the process, may remain hidden, e.g., the conformational heterogeneity of the denatured state [45, 46] and the transitions between intermediate conformations [47]. In these cases, another pair of collective variables, which reveal the corresponding elements of the reaction, can be used to see how the potential changes, similar as the FESs for different pairs of the variables are constructed to make the folding picture clearer [47, 48].

The basic components of the present approach are the calculation of the vector field of the probability fluxes in a two-dimensional space of orthogonal variables and its subsequent decomposition into divergence-free and curl-free fields. None of them is specific of protein folding. Therefore, the approach is potentially applicable to any complex reaction, for which the transition from the reactant to the product can be described by two (collective) variables.
